# Effects of intrarenal afferent stimulation by bradykinin on renal sympathetic nerve activity: tonic inhibition contributing to renal function

**DOI:** 10.1007/s00424-025-03116-1

**Published:** 2025-09-11

**Authors:** Eva Hutter, Tilmann Ditting, Martin Hindermann, Karl F. Hilgers, Roland E. Schmieder, Christian Morath, Mario Schiffer, Kerstin Amann, Roland Veelken, Kristina Rodionova

**Affiliations:** 1 Department of Internal Medicine 4 - Nephrology and Hypertension, Friedrich-Alexander University Erlangen, Ulmenweg 18, 91054 Erlangen, Germany; 2 Department of Internal Medicine 4 - Nephrology and Hypertension, Paracelsus Medical University Nuremberg, Nuremberg, Germany; 3 Department of Pathology, Friedrich-Alexander University Erlangen, Erlangen, Germany

**Keywords:** Bradykinin, Renal innervation, B2 receptors, TRPV1, Renal function, Sympathoinhibition

## Abstract

Bradykinin (BK) may increase renal sodium excretion by decreasing tubular ENaC activity. Afferent renal nerve activity (ARNA) putatively controls renal sympathetic nerve activity (RSNA) involved in renal sodium handling. We recently found tonic sympatho-inhibition due to intrarenal ARNA stimulation by the TRPV1 agonist capsaicin (CAP). Since BK is known to augment TRPV1 effects, we hypothesized that intrarenally applied BK also tonically inhibits RSNA. Four groups of rats (n = 8; BK, CAP, HOE + BK, NaCl-control) were equipped with arterial and venous catheters for blood pressure (BP) and heart rate (HR) recordings and drug application; bipolar electrodes for RSNA and ARNA recordings, renal arterial catheter for intrarenal administration (IRA) of bradykinin (BK: 10^−5^ M, 20 µl and 10^–4^ M; 2.5, 5, 10 µl), capsaicin (CAP 3.3, 6.6, 10 and 33*10^−7^ M, 10 µl). The B2-receptor antagonist HOE-140 (10^–4^ M, 40 µl) was administered intravenously (IV) just before IRA BK (HOE + BK), finally the NK_1_-receptor blocker RP67580 (10^−2^ M, 15 µl; IV) was applied in all groups at the end of the experiment. IRA BK and CAP momentarily increased ARNA. IRA CAP, IRA BK, and IRA HOE + BK, decreased RSNA from 4.2 ± 0.8 to 1.3 ± 0.2 µV*sec (BK, *P* < 0.01), 3.6 ± 0.5 to 0.9 ± 0.2 µV*sec (CAP, *P* < 0.01) and 3.2 ± 0.3 to 0.8 ± 0.1 µV*sec (HOE-BK, *P* < 0.01). Suppressed RSNA (BK, CAP, HOE + BK) was unmasked by IV RP67580: 1.6 ± 0.5 to 8.6 ± 2.9 µV*sec (BK, *P* < 0.01); 1.0 ± 0.2 to 6.1 ± 1.5 µV*sec (CAP, *P* < 0.01); 0.8 ± 0.2 to 4.5 ± 0.8 µV*sec (HOE-BK, *P* < 0.05). IRA BK was associated with momentary increases of RSNA, abolished by HOE-140. Intrarenal stimulation of renal afferent nerves by BK induced tonic renal sympathodepression likely augmenting sodium and water excretion.

## Introduction

Despite the negative outcome of the Simplicity 3 trial [6] recent well-controlled clinical studies have renewed interest in renal nerve ablation as a potentially effective treatment for arterial hypertension [10, 13, 18, 20, 21, 26, 33, 36, 39, 53]

The precise mechanisms by which renal innervation influences autonomic outflow remain incompletely understood [18, 36]. However, afferent renal nerves are increasingly recognized as crucial components in this regulation [2–4, 14, 32, 35]. This view is supported by reports of hypertensive patients with end-stage renal disease undergoing bilateral nephrectomy, which often results in a significant reduction of sympathetic activity [11, 22]. While earlier studies primarily attributed sympatho-excitatory properties to renal afferents in cardiovascular disease [20, 51, 52, 55] this perspective may be incomplete.

However, even intrarenal administration of bradykinin has been associated with enhanced sympathetic activity via renal afferent fibers [5]. This is notable, given that bradykinin in the renal interstitium primarily activates the B2 receptor [8] [7]. Inhibition of this receptor with icatibant (HOE-140) in perfused rat kidneys reduced urinary sodium excretion—presumably via modulation of ENaC activity in distal tubules—without affecting glomerular filtration rate (GFR) or renal blood flow [34, 47]. These effects mirror those observed during decreased renal sympathetic nerve activity (RSNA) [15, 25].

Our experimental work consistently supports a predominantly sympathoinhibitory function of renal afferent innervation, which appears attenuated in pathological states including hypertension and glomerulonephritis [31, 37, 43–45]. In a rat model of mesangioproliferative nephritis, RSNA was significantly elevated, while afferent signaling was markedly reduced [45]. Electrophysiological recordings from dorsal root ganglia (Th12–L2) with Renal afferents revealed a lower fraction of highly active neurons in nephritic animals in response to electrical stimulation. Comparable findings were obtained in rats with 2K1C renovascular hypertension [44]: afferent neurons innervating the stenotic kidney showed decreased excitability. Remarkably, renal denervation normalized this activity, suggesting that the removal of pathological renal signals unmasked the intrinsic excitability of afferent neurons remaining alive in the dorsal root ganglia.

In an earlier study, we demonstrated long-lasting sympathoinhibition following selective activation of TRPV1 receptors in the kidney via multiple bolus injections of capsaicin, a potent TRPV1 agonist [16]. This effect was found to be tachykinin dependent. Given that bradykinin can also activate TRPV1 through lipoxygenase-derived metabolites [48], we hypothesized that repetitive intrarenal bradykinin injections could reproduce the tonic sympathoinhibition observed with capsaicin [16].

Importantly, bolus injections likely result in a significantly lower cumulative bradykinin dose than continuous infusion (as in Barry et al. [5]), possibly avoiding the activation of nociceptive fibers such as those in the renal capsule, which may counteract any sympathoinhibitory effect [38].

To test these assumptions, we used an established catheter-based system for direct renal arterial injection of bradykinin in anesthetized rats while continuously monitoring arterial blood pressure (BP), heart rate (HR), and renal sympathetic nerve activity (RSNA). Specific B2 and tachykinin receptor antagonists were administered to delineate the pathways involved in the observed autonomic responses.

## Material and methods

### Animals

Male Sprague–Dawley rats (Charles River, Kisslegg, Germany) weighing 250 to 300 g were maintained in cages at 24 ±  2 °C and fed a standard rat diet (No. C-1000, Altromin, Lage, Germany) containing 0.2% sodium by weight and were allowed free access to tap water. All of the procedures performed on animals were done in accordance with the National Institutes of Health Guide for the Care and Use of Laboratory Animals and approved by the local government agency (Regierung von Mittelfranken, Ansbach, Germany).

#### Anesthesia

For all surgical procedures, animals received preemptive analgesia with buprenorphine (0.05 mg/kg, s.c.) and were anesthetized using a mixture of O₂ and approximately 3% isoflurane. Upon completion of the surgical procedures, anesthesia was switched to thiopental 120 mg/kg (Trapanal, Inresa GmbH, Freiburg, Germany) administered intravenously at a rate of 0.1 ml per minute. Animals were then allowed to stabilize for 90 min. The thiopental maintenance infusion was adjusted according to the depth of anesthesia, assessed by withdrawal reflexes, palpebral and corneal reflexes, and respiratory rate.

#### Arterial and venous lines and bladder catheter

A right-sided femoral artery catheter (PE-10 tip attached to PE-50 tubing; PE-10/50) was connected to a Stratham P23Db transducer to record arterial blood pressure and heart rate (pressure processor type 13–4615-52; Gould Instrument Systems, Valley View, OH). Two right-sided femoral venous lines (PE-10/50) were inserted for intravenous (IV) administration of substances and for methohexital maintenance infusion. Another catheter (PE-10/50) was placed in the right jugular vein to monitor the right atrial pressure and the respiratory rate and for saline infusion (1.3 ml/kg per hour).

Via a caudoventral and longitudinal abdominal incision (< 1 cm) the bladder was exposed extraperitoneally and a polyethylene catheter (OD: 1.7 mm; ID: 1.3 mm) with a silastic cuff was implanted and fixed with sutures to prevent urinary retention and consecutive sympathetic excitation due to potential anesthesia side effects.

#### Renal artery catheter

The renal artery catheter system was assembled as previously described [16]. Briefly, the tip of a PE-10 catheter (OD: 0.61 mm; ID: 0.28 mm) was heated and stretched to an outer diameter (OD) of 120 µm over 10 mm length. This heat-stretched tip was coiled around a glass rod with a diameter of 1.2 mm, followed by immersion in hot water (80°C) for 5 s and immediate cooling in ice water. The resulting coil was cut in such a way that a 90° curve with a radius of 0.6 mm was obtained. This curved microtip catheter was inserted into a vascular introducer sheath made from an intravascular infusion catheter (22G, Insyte-W. Becton Dickinson, Germany) of which the Luer-cone was assembled with a silastic valve to prevent bleeding while positioning the catheter-tip. The catheter system was inserted into the left femoral artery and advanced up the aorta to position the tip just 2 mm cranial to the ostium of the left Renal artery. Then, the left kidney was exposed via a left lateral incision and carefully retracted ventrally while the rat was lying on its right side. The curved microtip catheter was manipulated to advance the tip 1.0 to 1.5 mm into the left renal artery to be then used for the injection of substances without occluding the renal artery.

#### Recording of renal sympathetic nerve activity (RSNA) and afferent renal nerve activity (ARNA)

Recordings of left-sided ARNA or right-sided RSNA were performed in separate experiments as previously described [16, 17, 28, 29]. Through a right-sided dorsolateral, or left lateral approach respectively, a renal nerve bundle was dissected free from connective tissue and placed on a bipolar electrode (0.2-mm stainless steel wire, Science Products, Frankfurt, Germany). RSNA was recorded from a proximal right-sided renal nerve branch (contralateral to drug administration), while ARNA was recorded from the distal cut end of a left-sided renal nerve filament (ipsilateral to drug administration)**.”**RSNA and ARNA signals were amplified 50.000 times and filtered (1 kHz low pass; 100 Hz high pass) using a band pass amplifier (CyberAmp 320 with an AI402 × 50 Ultra Low Noise Differential amplifier; Axon Instruments, Foster City, CA, USA).

Respective signals were channeled to an A/D oscilloscope (HM 305–3; Hameg, Frankfurt, Germany) and an audio amplifier (AM8 audio monitor; Grass-Telefactor, West Warwick, RI, USA) for visual and auditory evaluation. RSNA signal quality was assessed by its pulse synchronous rhythmicity and by the reversible disappearance of bursting multifiber activity due to ganglionic blockade by trimetaphan-camsylate (ARF 10 mg/kg IV; Arfonad®; Roche, Basel, Switzerland). When an optimal signal could be observed the right-sided nerve bundle was fixed to the electrode using silicone adhesive (Bisico S4i; Bielefelder Dentalsilicone, Bielefeld, Germany).

When the left-sided distal nerve filament (ARNA) was cut proximal to the electrode, bursting activity disappeared right away and irreversibly, but single spontaneous action potentials became visible. The left-sided distal cut end of the renal nerve filament was likewise fixed to the electrode with silicone adhesive. Respective electrodes were tunneled to the neck, and the situs was closed in layers.

The nerve signals were full-wave rectified and integrated over 1-s intervals using a commercially available data acquisition and analysis software (SciWorks 7.2, DataWave Technologies, Loveland, CO, USA). At the end of the nerve recording protocol ARF and KCl were given to evaluate baseline noise as well as maximum depolarized nerve bundle activity.

The RSNA recording experiments commenced with baroreceptor loading and unloading, by IV bolus administration of the α_1_-agonist methoxamine (METH 10µg) and the vasodilator Na^+^-nitroprussid (NIP 1µg), respectively, in randomized order with a recovery period of 10 min, to confirm a well-balanced resting level of RSNA at the beginning of the recording period.

#### Experimental protocols

##### Bradykinin (BK) dose–response experiments

**Fig. 1:**
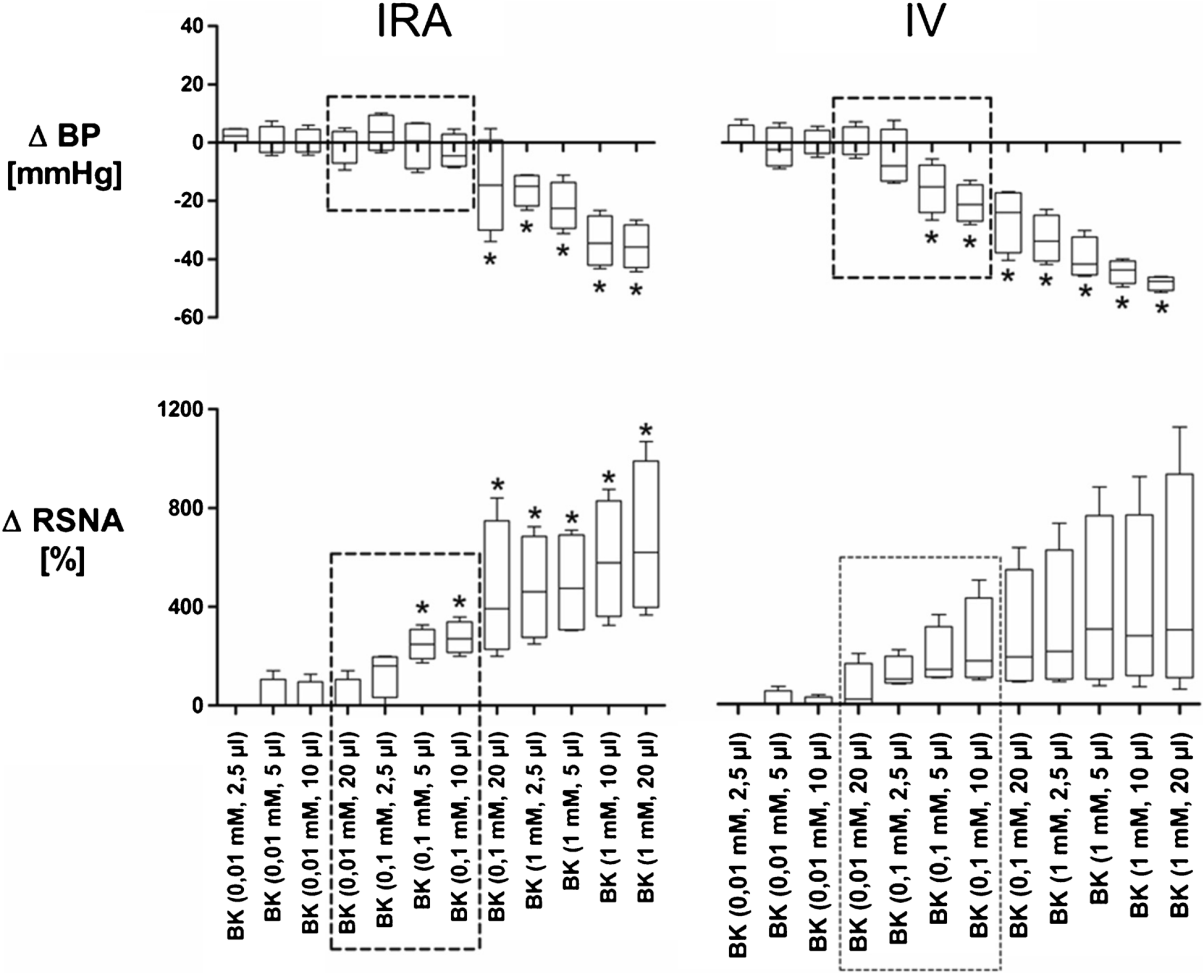
Dose-dependent changes (∆) of blood pressure (BP) and contralateral renal sympathetic nerve activity (RSNA) due to ipsilateral intrarenal (IRA) and systemic (IV) application of increasing bradykinin doses (P < 0.05) measured during the first 3–5 s. The highlighted (boxed) doses are the ones with minor or no effect on BP but with obvious increase in RSNA that were chosen for the following experiments

In this preliminary experiment twelve increasing doses of BK (1 × 10^–5^ M, 2.5 µl to 1 × 10^–3^ M, 20 µl; see Fig. 1) were applied either intrarenally (IRA) or systemically (IV) in two separate groups of rats (n = 4, each) while blood pressure (BP), heart rate (HR), and RSNA were recorded. These experiments were done to determine those putative doses of BK that might induce even subtle changes in RSNA (in any direction), while not affecting the blood pressure. Since BK acts as a potent vasodilator, unloading of arterial baroreceptors would inevitably induce reflex RSNA activation that will mask putative “kidney specific” BK effects. Thus, these experiments aimed to clarify if BK-dependent effects specifically mediated by renal afferent nerves do exist, and if so, whether they would be detectable by the IRA application method we used.

##### ARNA response due to intrarenal application of bradykinin (BK) and capsaicin (CAP)

Rats (n = 6) were instrumented with arterial and venous catheters, a left-sided renal arterial (IRA) catheter and left-sided electrode for ARNA recording. Bolus injections of submaximal doses of BK (1 × 10^–4^ M, 5µl—as established in the first experimental protocol, see Fig. 1) and CAP (1 × 10^–6^ M—as established previously [16]) were intrarenally administered (IRA) to assess the effects on ARNA, blood pressure and heart rate. This experiment aimed to clarify if both CAP and BK may induce similar ARNA responses.

##### Renal sympathetic responses due to intrarenal administration of BK and CAP

Three groups of rats (n = 8) were equipped with arterial and venous lines. Furthermore, they were equipped with a left-sided renal arterial catheter (ipsilateral kidney) and a right-sided (contralateral kidney) electrode. In these three different groups of rats increasing doses of either bradykinin (BK 1 × 10^–5^ M, 20 µl; 1 × 10^–4^ M, 2.5, 5, 10 µl) or Capsaicin (CAP 3.3 × 10^–7^ M, 6.6 × 10^–7^ M, 1 × 10^–6^ M, 3.3 × 10^–6^ M, 10 µl each) or normal saline (NaCl 0.9%, 10µl) were applied intrarenally (IRA) at intervals of 15 min, similar to the protocol previously described [16]. The responses of RSNA, mean arterial blood pressure (MAP) and heart rate (HR) were assessed. This protocol was done to determine if intrarenally applied BK also tonically decreases RSNA as previously described for intrarenally applied capsaicin [16], according to the hypothesis described in the introduction.

##### Administration of the Bradykinin B2 receptor antagonist HOE-140 [9]

In a fourth group of rats (n = 8) equipped as described above (see protocol 3), the bradykinin B2-receptor antagonist HOE-140 (10^–4^ M, 40µl) was administered intrarenally (IRA) just before the first bradykinin bolus application in order to assess the role of BK receptors in the responses due to intrarenal BK application. In preliminary experiments the action time of HOE-140 was established. We found that one single dose of HOE-140 applied just before the first BK-bolus reliably suppressed the BK induced RSNA-response of the four consecutive BK bolus applications given at an interval of 15 min each (T0´, T15´, T30´, T45´), while T0´ was defined as the point in time when the first bolus dose of the respective IRA test substances was applied after stable baseline parameters were obtained; see Fig. 4).

##### Administration of the tachykinin NK1 receptor antagonist RP67580 [23]

In all animals described in protocol 3 and protocol 4, the NK1-receptor antagonist RP67580 (10^–2^ M, 15µl IV) was applied 75 min (T75´) after the first IRA bolus dose of the respective test substances (NaCl 0.9%, or BK, or BK-HOE, or CAP) to block NK1-receptors, since we assumed that a tachykinin dependent mechanism might be involved in the RSNA responses due to intrarenal administration of bradykinin, as shown for capsaicin before [16].

### Data analysis

Integrated RSNA and ARNA were recorded in microvolts*seconds (µV*sec). To compare baseline values between groups, individual values were corrected for background noise, which is the postmortem activity (30-min-average) and/or the maximal response due to intravenous Arfonad®. Furthermore, recorded data were corrected for amplifier gain (50.000x). Baseline values of RSNA (µV*sec), mean arterial pressure (MAP [mmHg]) and heart rate (HR [bpm]) were averaged over 30 s before each intervention (i.e. application of test substances). All data were tested for normality using the Kolmogorov–Smirnov-test. Non-parametric tests were used when data failed normality testing. This was the case only for the preliminary BK-dose–response experiment, where the Mann–Whitney Rank Sum Test was used for statistical analysis.

Baseline parameters and differences between groups at single points in time were analyzed using One-Way-ANOVA with Student–Newman–Keuls post hoc “all pairwise comparison”. Changes of parameters over the time within groups were tested with One-Way Repeated Measures ANOVA with Dunnett post hoc “multiple comparisons versus control” (ie, baseline).

Statistical significance was defined as *P* < 0.05. Data are given as group means ± SEM. Not normally distributed data are shown as standard Box Plots. GraphPadPrism (GraphPad Software Inc., La Jolla, CA, USA) was used for statistical analysis.

## Results

### Bradykinin (BK) related dose–response experiments

To determine those IRA BK doses with negligible blood pressure effect, but with obvious but short-lived increases in RSNA, a series of dose–response experiments was done (see Fig. 1).

Twelve increasing concentrations of BK (10^−5^M 2.5 µl to 10^–3^ M, 20 µl) were given either intrarenally or systemically/intravenously (n = 4 each). Being a potent vasodilator, BK caused significant but short-lived decreases in blood pressure after intrarenal (0.1 mM, 10^–4^ M, 20 µl and higher) and systemic (0.1 mM, 10^–4^ M, 5 µl and higher) application (*P* < 0.05). Lower concentrations showed no significant BK-dependent changes in blood pressure. Both intrarenal and systemic applications of BK showed dose-dependent RSNA increases. In contrast to the systemic administration, intrarenal application of BK (0.1 mM, 5 µl and above) caused significant RSNA increases (*P* < 0.05) even in this small sample size. When given IV, BK tended to increase RSNA only if blood pressure was decreased indicating a non-specific effect probably due to unloading arterial baroreceptors. This effect was more variable and failed to reach statistical significance most likely due to the small sample size.

In fact, there seemed to be some doses of BK that increased RSNA without affecting blood pressure when given intrarenally, while some of these doses already decreased blood pressure when given systemically (IV). Thus, some kind of “kidney-specific” bradykinin effect supposably mediated by afferent renal nerves seems to exist. Based on these dose–response experiments the highlighted (boxed) four doses of intrarenal bradykinin (0.01 mM, 20 µl and 0.1 mM 2.5, 5, 10 µl, see Fig. 1) were chosen for further experiments.

### ARNA response due to intrarenal application of bradykinin (BK) and capsaicin (CAP)

**Fig. 2:**
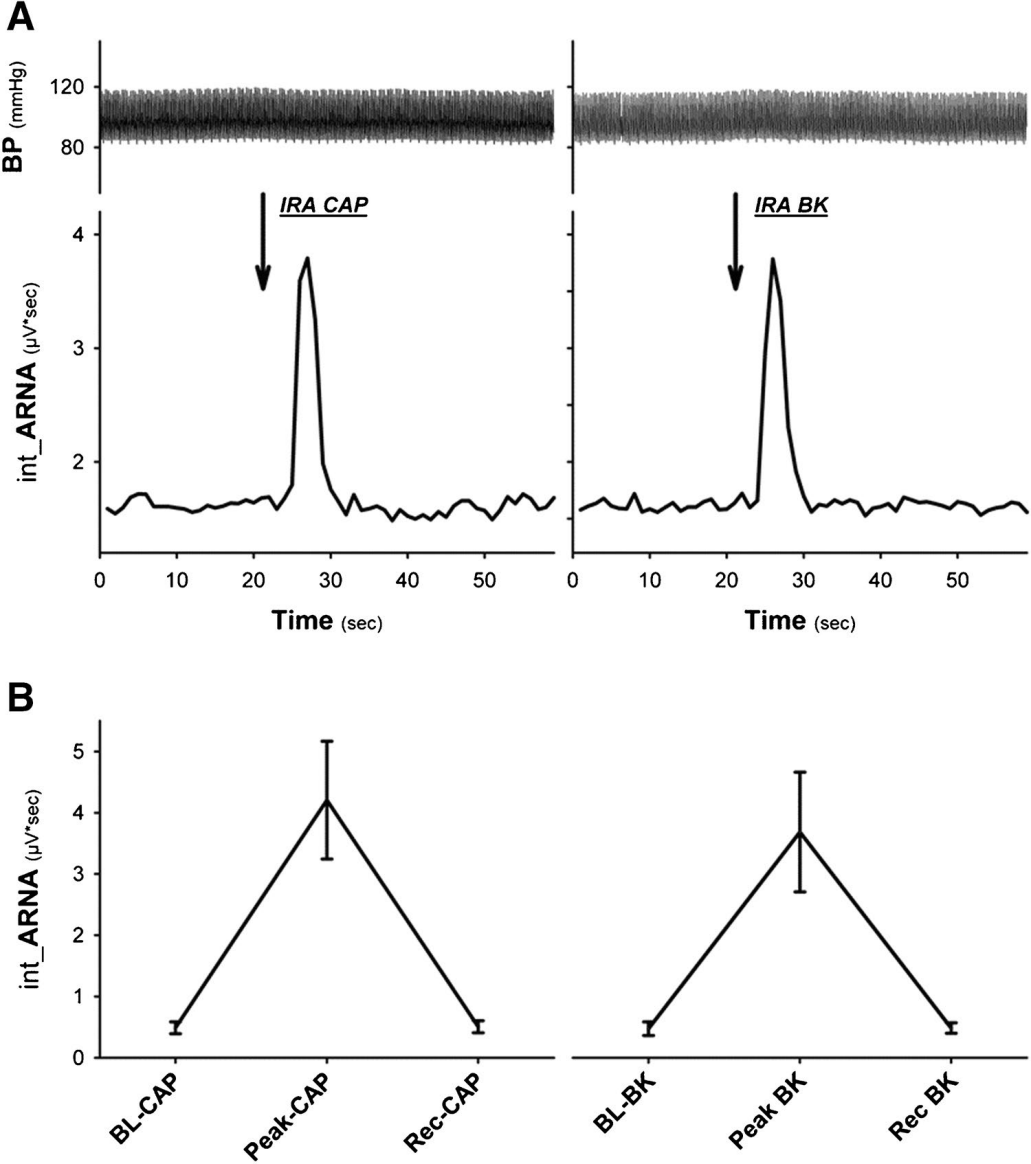
Panel A Original data traces of blood pressure (BP) and integrated afferent renal nerve activity (int_ARNA; contralateral kidney) showing the response due to intrarenal (IRA) application of either capsaicin (CAP, left side) or BK (right side) into the ipsilateral kidney. Panel B: IRA CAP and IRA BK induced similar short-lived but significant activation of ARNA (n = 6; P < 0.05, Peak vs. baseline BK & recovery Rec)

Intrarenal stimulation of afferent renal nerve activity [16] by injection of capsaicin (IRA CAP; 6.6*10-7M) into the renal artery elicited a short-lived but strong increase of ARNA (see Fig. 2). Comparing this response to the effect of intrarenal bradykinin application (IRA BK; 1 × 10^–4^ M, 5µl) showed a very similar ARNA response (see Fig. 2).

The integrated ARNA values (~ 0.5 µV·s⁻^1^) in our setup reflect the spontaneous spike frequency of afferent fibers after ganglionic blockade, not tonic voltage changes. In contrast to RSNA, which includes compound efferent bursts synchronized to the cardiac cycle, ARNA consists of irregular spike trains whose temporal density contributes to the integrated value. This physiological difference explains the lower absolute ARNA values and their interpretation as spike frequency rather than continuous signal power.

### Long-term sympatho-inhibition due to intrarenal stimulation of peptidergic afferents

**Fig. 3:**
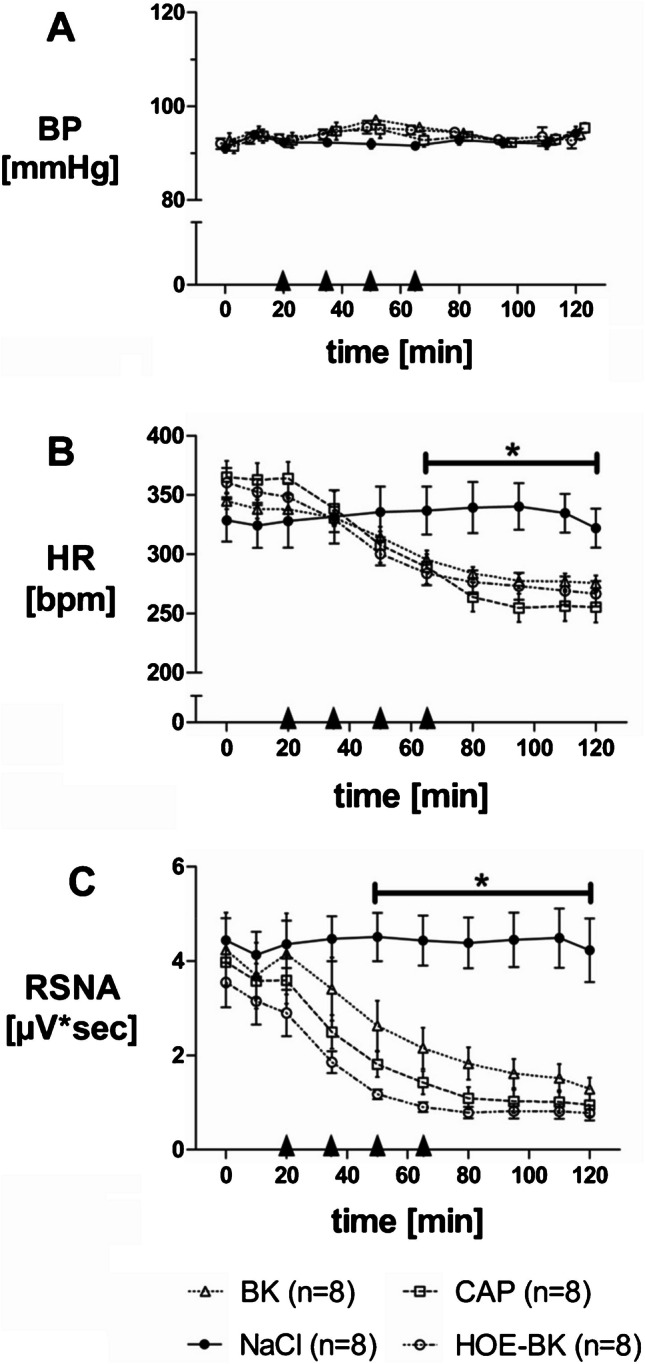
Long-term effects of Bradykinin (BK), Bradykinin with HOE-140 pre-treatment (HOE-BK), Capsaicin (CAP) and normal saline (NaCl) on blood pressure (BP, A), heart rate (HR, B) and renal sympathetic nerve activity (RSNA, C). Panel A: Application of BK, HOE-BK, CAP or NaCl did not change BP over time in any group. Panel B: HR decreased due to administration of BK, HOE-BK and CAP compared to normal saline control (P < 0.05). Panel C: RSNA decreased due to administration of BK, HOE-BK and CAP compared to normal saline control (P < 0.05)

After determining four IRA BK doses with negligible blood pressure effects, these doses were given consecutively at intervals of 15 min, (arrow heads in Fig. 3; T0´, T15´, T30´, T45´) into the ipsilateral renal artery (BK-group).

In the HOE-BK group one single bolus of the B2-receptor blocker HOE-140 was applied just before the first dose of BK. Normal saline (NaCl control group) and the previously established [16] doses of the TRPV1 agonist capsaicin (CAP group) were used as negative and positive controls respectively. Within each group, intrarenal application of BK, HOE-BK or CAP did not cause significant changes in blood pressure (Fig. 3A, overall means: BK 94.0 ± 1.6 mmHg; HOE-BK 93.6 ± 1.1 mmHg; CAP 93.4 ± 1.3 mmHg; NaCl 92.5 ± 1.0 mmHg).

Within the first 20 min heart rate did not show any significant difference between the four groups (Fig. 3B, means 0–20 min: BK 340.3 ± 4.4 bpm; HOE-BK 353.6 ± 6.4 bpm; CAP 363.8 ± 7.7 bpm; NaCl 326.8 ± 10.9 bpm). Consecutive applications of BK, HOE-BK and CAP (arrow heads in Fig. 3, i.e t0´, t15´, t30´, t45´´) caused a slow but significant decrease of heart rate compared to the normal saline control group (Fig. 3B, means 65–120 min: BK 281.8 ± 2.8 bpm, HOE-BK 273.7 ± 4.7 bpm, CAP 263.7 ± 5.8 bpm *vs.* NaCl 334.9 ± 8.2 bpm; *P* < 0.05). During the first 20 min renal sympathetic nerve activity (RSNA) of the contralateral kidney did not show any significant difference between the four groups (Fig. 3C, means 0–20 min: BK 4.0 ± 0.4 µV*sec; HOE-BK 3.2 ± 0.3 µV*sec; CAP 3.7 ± 0.3 µV*sec; NaCl 3.7 ± 0.4 µV*sec). Repeated administration of BK, HOE-BK and CAP (arrow heads in Fig. 3C) induced a slow but significant inhibition of RSNA when compared to the normal saline control group (means 95–120 min: BK 1.5 ± 0.2 µV*sec, HOE-BK 0.8 ± 0.1 µV*sec, CAP 1.0 ± 0.1 µV*sec *vs.* NaCl 3.3 ± 0.4 µVᵡsec, *P* < 0.05).

### Bradykinin (BK)-dependent short-lived changes

**Fig. 4:**
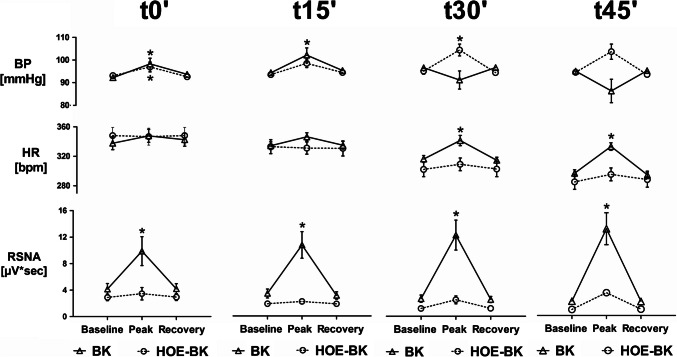
Short-term effects of BK and HOE-BK on blood pressure (BP, upper row), heart rate (HR, middle row) and renal sympathetic nerve activity (RSNA of the contralateral kidney, lower row). Upper row: The lower doses of BK increased BP in the BK- and the HOE-BK group. The higher doses of BK tended to decrease BP in the BK group but increased BP in the HOE-BK group. Middle row: Only the higher BK doses increased HR significantly within the BK group. All BK doses were without effect on heart rate within the HOE-BK group. Lower row: RSNA was increased by BK in dose-dependant matter. This was completely abolished by HOE-140 pre-treatment. (* P < 0.05, Baseline vs. Peak vs. Recovery)

Beside the long-term effects, intrarenally applied bradykinin (BK) (ipsilateral kidney) also caused short-term changes in mean arterial blood pressure (MAP), heart rate (HR) and renal sympathetic nerve activity (RSNA) of the contralateral kidney (see Fig. 4). Administration of the lowest BK dose (1 × 10^–5^ M, 20 µl) caused a very small but significant short-lived increase in MAP (t0´ BK: 92.2 ± 0.9 mmHg to 98.3 ± 2.5 mmHg;* P* < 0.05). The second BK dose (1 × 10^–4^ M, 2.5 µl; Fig. 4, upper row, t15´) also induced a small MAP increase (t15´ BK: 94.8 ± 0.7 mmHg to 102.8 ± 3.2 mmHg, *P* < 0.05). However, the two highest BK-doses rather tended to decrease MAP (t30´ BK and t45´BK, Fig. 4 upper row).

Within the HOE-BK group, MAP also increased slightly with the first BK dose (t0´ BK-HOE: 93.1 ± 0.9 mmHg to 96.9 ± 2.1 mmHg; *P* < 0.05), as it did with the second (1 × 10^–4^ M, 5 µl, t15´ BK-HOE; 94.9 ± 1.0 mmHg to 104.4 ± 3.5 mmHg; *P* < 0.05) and with the third (t30´ HOE-BK: 94.9 ± 1.0 mmHg to 104.4 ± 3.5 mmHg, *P* < 0.05). The highest BK dose (1 × 10^–4^ M, 10 µl; t45´ BK-HOE) also increased MAP (see Fig. 4, upper row) but failed significance testing.

After the initial HOE-140 pre-treatment dose all four concentrations of BK (BK-HOE) were without effect regarding heart rate (HR) (Fig. 4, middle row). In absence of HOE-140, however, the two highest doses of BK induced significant HR increases (1 × 10^–4^ M, 5 µl; t30´ BK: 314.3 ± 4.7 bpm to 338.9 ± 6.9 bpm, *P* < 0.05; 1 × 10^–4^ M, 10 µl, t45´ BK: 295.5 ± 4.6 bpm to 331.3 ± 5.1 bpm, *P* < 0.05). This effect was completely blocked by HOE-140.

In the context of RSNA the effects were even more pronounced: After the initial HOE-140 pre-treatment RSNA did not increase due to BK application (Fig. 4, lower row). However, without HOE-140 pre-treatment IRA BK induced strong and seemingly dose-dependent but rather short-lived RSNA increases. The first BK dose (1 × 10^–5^ M, 20 µl, lower row, t0´ BK) increased RSNA from 4.2 ± 0.9 µV*sec to 9.9 ± 2.2 µV*sec (*P* < 0.05). The second BK dose (1 × 10^–4^ M, 2.5 µl, t15´ BK) increased RSNA from 3.4 ± 0.7 µV*sec to 10.7 ± 2.0 µV*sec, *P* < 0.05. The third BK dose (1 × 10^–4^ M, 5 µl, t30´ BK) increased RSNA from 2.6 ± 0.5 µV*sec to 12.1 ± 2.2 µV*sec, *P* < 0.05. Finally, the highest BK dose (1 × 10^–4^ M, 10 µl, t45´ BK) increased RSNA from 2.1 ± 0.4 µV*sec to 13.1 ± 2.4 µV*sec *P* < 0.05. This BK induced short-lived RSNA excitation observable at any RSNA level was completely abolished by the pre-treatment with the specific B2-receptor antagonist HOE-140 (whereas t0´ denotes baseline, t15´ to t45´ denote progressive suppression levels).

### Re-activation of tonically suppressed RSNA by NK1-antagonism

**Fig. 5:**
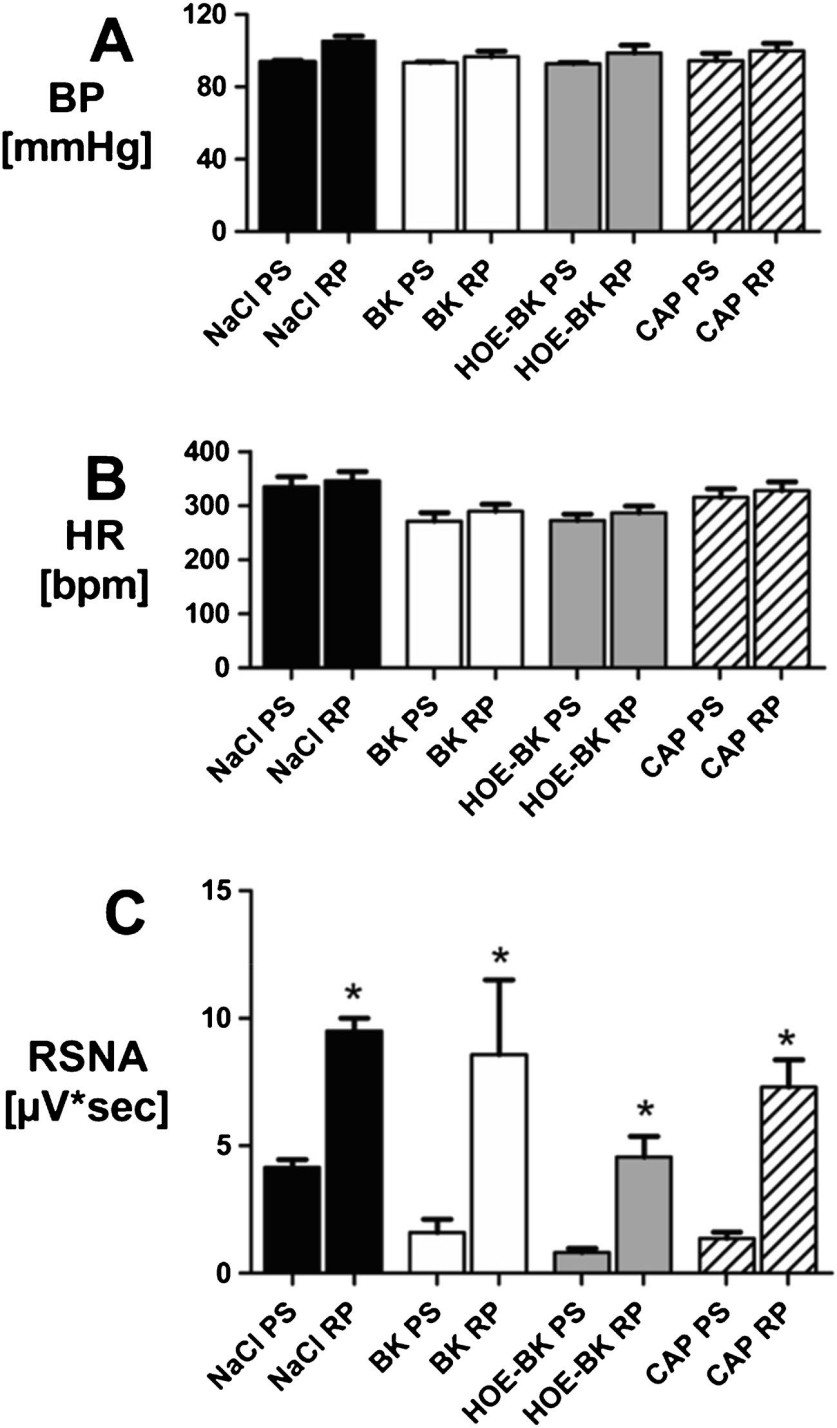
Short-term changes in blood pressure (BP, A), heart rate (HR, B) and renal sympathetic nerve activity (RSNA, C) due to systemic (IV) administration of NK1-receptor antagonist RP67580 (1 × 10^–2^ M, 15 µl). Comparison of pre-stimulus (PS) and RP67580 (RP) values. Panel A, B: NK1-antagonism had no significant effect on either BP or HR in any of the four groups. Panel C: RP67580 caused increase of RSNA (P < 0.05) within all groups and thus more than complete reversal of the tonic RSNA suppression induced by CAP, BK and HOE-BK. In the NaCl control group RSNA was also increased, indicating tonic RSNA suppression already under normal conditions. (*P < 0.05, pre-stimulus (PS) vs. RP67580 (RP))

After demonstrating capsaicin- and bradykinin-induced tonic RSNA suppression (see Fig. 3C) it remained to be determined if the BK induced tonic RSNA suppression could be abolished by NK1-antagonism, as previously shown for capsaicin induced tonic RSNA. suppression [16]. We therefore systemically (IV) administrated the NK1-receptor antagonist RP67580 (RP, 1 × 10^–2^ M, 15 µl, IV) 75 min (t75´) after the first application of the respective test substances (BK, BK-HOE, CAP, NaCl, see Fig. 5). Administration of RP67580 (RP) did not show relevant changes in blood pressure in any group (Fig. 5A). There was also no effect on heart rate (Fig. 5B). However, RP67580 strongly increased RSNA from baseline (saline control group NaCl) and also from suppressed level in all other groups (Fig. 5C).

## Discussion

In line with our working hypothesis, systemic administration of bradykinin resulted in a sustained decrease in renal sympathetic nerve activity (RSNA), which could be reversed or even exceeded baseline levels by intravenous application of a tachykinin NK1 receptor agonist. In this respect, bradykinin exerted a functionally similar effect to capsaicin, a direct TRPV1 receptor agonist, as previously demonstrated in a separate series of experiments [16]. These observations reinforce prior evidence suggesting that bradykinin may act indirectly on sympathetic output by interacting with the TRPV1 receptor system [30, 48]. Our findings provide further support for the notion that afferent renal innervation plays a significant role in a predominantly sympathoinhibitory regulatory network [31, 37, 43–45].

This study expands upon earlier results by demonstrating that the afferent renal system includes a tonic, NK1-dependent mechanism that actively inhibits sympathetic outflow. The long-lasting suppression of RSNA induced by both bradykinin and capsaicin could be reliably reversed by systemic administration of the selective NK1 receptor antagonist RP67580 [16], a well-characterized non-peptide compound with high receptor specificity [22].

These sympathoinhibitory effects appear to arise from neuro-paracrine mechanisms—presumably through the release of neurokinins from afferent renal nerves—rather than from increased afferent activity per se. In fact, direct recordings of renal afferent nerve activity following intrarenal capsaicin injections revealed only short-lasting bursts of action potentials, which bore no temporal correlation to the prolonged suppression of sympathetic tone observed subsequently [15]. Thus, the data suggest that neurogenic modulation of sympathetic activity was also dependent on substance P (SP) contained within afferent nerve fibers [42], likely influencing in a paracrine fashion classic neural signaling.

Such transmitter release may not be confined to renal nerve terminals. Afferent peptidergic fibers have been shown to release potentially along their entire course of the afferent pathway, suggesting that sympatho-afferent interactions could occur within peripheral autonomic ganglia [54]. Though we have yet to document such interactions directly, immunohistochemical evidence supports this possibility. Peptidergic afferent fibers traveling through the aortico-renal ganglion were observed in proximity to sympathetic neurons originating from the kidney [16], offering a plausible anatomical substrate for functional interaction between afferent and efferent fibers. Such interactions might extend beyond the renal system and could contribute to broader autonomic regulation.

The proximity of afferent and efferent fibers in the aortico-renal ganglion supports the possibility of local interactions as reported earlier [16]. In addition, the suppression of contralateral RSNA indicates also a further role for central processing of renal sympathetic innervation via central pathways [46].

Interestingly, the observed sympathoinhibition following repeated intrarenal TRPV1 stimulation was not accompanied by significant changes in arterial blood pressure or heart rate [16], suggesting that this mechanism is largely restricted to renal efferent pathways. However, in our current experiments, both capsaicin and bradykinin produced small but consistent decreases in heart rate, implying some systemic effect, possibly via central integration of renal afferent input.

TRPV1 receptors are known to be expressed exclusively on peptidergic afferent neurons [27, 49, 56], which project to the dorsal root ganglia [19, 40]. Therefore, the observed responses can be attributed specifically to this subset of afferent fibers. This likely anatomical specificity strengthens the argument that the observed renal sympathoinhibition is mediated by a well-defined neurogenic pathway involving TRPV1 and NK1 receptors.

In contrast to the long-term inhibitory effects, we also observed short-lived increases in RSNA following bradykinin administration. These transient responses were abolished by the B2 receptor antagonist HOE-140 in a dose-dependent fashion, indicating that B2 receptor activation contributes to an initial sympathoexcitatory phase. While the precise mechanisms remain unclear, it could be possible that nociceptive afferents from the renal capsule—which is the only structure in the kidney known to mediate pain—are involved. B2 receptors are involved in neurogenic pain pathways [24], so that afferent fibers in the capsule may transmit excitatory signals upon bradykinin stimulation.

The preserved long-term RSNA suppression in the HOE-BK group may reflect a shift from acute B2 receptor-mediated excitation to unopposed TRPV1-dependent inhibition. BK can activate TRPV1 indirectly via lipoxygenase metabolites [1], and such mechanisms may remain intact despite B2-receptor blockade.

We did not record continuous ARNA during repeated BK or CAP administration. However, based on previous findings [16], we hypothesize that sustained afferent discharge is not necessary for long-term RSNA inhibition. Rather, the prolonged effect appears to rely on peptidergic transmitter release (e.g., substance P) from afferent terminals, acting via NK1 receptors in a paracrine manner.

We have previously described a bradykinin-induced cardio-renal reflex, in which intrapericardial application of bradykinin activated cardiac afferent fibers and increased blood pressure, whereas systemic application of the same dose produced hypotension [50]. This discrepancy suggests that the site of bradykinin action is critical in determining its physiological effects. Phenylbiguanide, a serotonin 5-HT3 receptor agonist that activates cardiac vagal afferents and induces sympathoinhibition, was used as a control agent [47]. The fact that bradykinin’s effect on blood pressure varied with the site of administration underscores the importance of differentiating between local and systemic pathways when interpreting neurogenic modulation[12]

Further studies have reported prolonged RSNA increases after bradykinin administration into the cortico-medullary junction of the kidney [5]. This does not contradict our findings but rather highlights the importance of experimental context. In that study, the high local concentration of bradykinin at the nephron level may have elicited both neurogenic and non-neurogenic responses, potentially involving local vascular effects. Bradykinin is known to induce vasodilation in the efferent arterioles downstream of glomeruli via cytochrome P450-derived metabolites [41], while higher doses can constrict the afferent arterioles upstream. It remains speculative whether a transient vasodilation during intrarenal bradykinin injection might trigger a reflexive sympathoexcitation via juxtavascular afferents. Answering this question would require more targeted experimental designs.

A plausible physiological implication is that a sequence of short-term RSNA increases might modulate renal sodium and water reabsorption, while more sustained TRPV1-dependent inhibition provides a counter-regulatory mechanism. The interplay of these dynamics may enable the kidney to finely tune excretory functions under both physiological and pathophysiological conditions [15].

### Perspective

TRPV1 receptors are modulated by a variety of endogenous compounds, many of which are upregulated in states of renal inflammation, fibrosis, or metabolic stress [30, 48]. The present study shows that bradykinin can induce sympathoinhibition via NK1 receptor activation downstream of TRPV1-positive afferents. This finding expands our understanding of the kidney’s afferent innervation as a critical regulator of systemic sympathetic tone. It suggests that the role of TRPV1 receptors in renal afferent signaling extends beyond nociception and may be integral to long-term autonomic and fluid homeostasis. Future research should explore the therapeutic potential of modulating this system in conditions such as hypertension, chronic kidney disease, and heart failure.

## Data Availability

No datasets were generated or analysed during the current study.
